# Absence of Long-Lasting Morning Stiffness at the Time of Diagnosis as Paraneoplastic Warning in Patients with Polymyalgia Rheumatica (PMR): Data from Italian Single-Center Study

**DOI:** 10.3390/life13061285

**Published:** 2023-05-30

**Authors:** Ciro Manzo, Alberto Castagna, Marco Isetta

**Affiliations:** 1 Internal and Geriatric Medicine Department, Rheumatologic Outpatient Clinic, Azienda Sanitaria Locale Napoli 3 Sud, Health District No. 59, Sant’Agnello, 80065 Naples, Italy; 2 Primary Care Department, Azienda Sanitaria Provinciale Catanzaro, 88068 Soverato, Italy; albertocastagna@tiscali.it; 3 Library and Knowledge Services, Central and North West London NHS Foundation Trust, London NW1 3AX, UK; marcoisetta192@gmail.com

**Keywords:** polymyalgia rheumatica, paraneoplastic syndrome, morning stiffness, single-center cohort study, observational study

## Abstract

Background: There is little literature on the paraneoplastic value of the absence of long-lasting morning stiffness (MS) at the time of diagnosis of polymyalgia rheumatica (PMR). We investigated whether and to what extent this finding was related to the probability of diagnosing a neoplasia. Patients and Methods: This was an observational, retrospective, single-center cohort study. We enrolled all patients consecutively referred to our rheumatologic outpatient clinic between January 2015 and December 2020, who could be classified as PMR according to 2012 EULAR/ACR criteria. In particular, we assessed all patients scoring a minimum of five points with a combination of clinical and ultrasound (US) criteria. The exclusion criteria were as follows: (a) follow-up duration <two years; (b) malignancy prior to PMR; (c) first-degree familiarity of malignancy; (d) incomplete data; and (e) diagnostic change during follow-up in different rheumatologic diseases. Results: 143 patients (108 women; median age: 71.5 years) were enrolled, and 35 of them did not have long-standing MS at the time of PMR diagnosis. In 10 patients (6.9%), a neoplasia was diagnosed in the first 6 months of follow-ups; among these, 7 did not have long-lasting MS. Among the remaining 133 PMR patients without subsequent malignancy, 28 did not have long-lasting MS. The odds of cancer were 0.114 (C.I. 95% 0.028, 0.471). Long-lasting MS was inversely associated with the development of neoplasias. In all eight PMR patients diagnosed with solid cancers during follow-ups, the removal of the neoplastic mass led to a fast disappearance of clinical, ultrasound and laboratory findings, thus supporting the diagnosis of paraneoplastic PMR. Finally, a positive response to glucocorticoids (GCs) was present in 100% of the 28 PMR patients without long-lasting MS at the time of diagnosis and without neoplasia during their follow-ups. On the contrary, a positive response to GCs was present in 71% of PMR patients without long-lasting MS and neoplasias during follow-up. Among the variables we assessed, a positive response to GCs was the only one that was statistically significant (*p* < 0.0001). These data suggested that an inadequate response to GCs in PMR patients without long-lasting MS at the time of diagnosis should strengthen investigations to rule out neoplasias. Conclusions: The absence of long-standing MS at the time of diagnosis can be a paraneoplastic warning in patients classified as PMR. A thorough investigation is therefore needed in this subset of patients to rule out neoplasia, before diagnosing an idiopathic PMR and starting treatment with GCs.

## 1. Introduction

The relationship between polymyalgia rheumatica (PMR) and neoplasias is still a matter of debate [1,2,3,4], and the results of studies searching for paraneoplastic warnings at the time of PMR diagnosis are not conclusive. For instance, some researchers published a systematic review of clinical studies on PMR as a paraneoplastic syndrome. They found little evidence to suggest what features may be associated with the development of cancer, and concluded that it was not possible to identify patients with potential paraneoplastic PMR [5]. On the other hand, studies from monocentric cohorts suggested that swollen joints and peripheral arthritis [6], or high C-reactive protein (CRP) concentrations at baseline [7], should be considered as paraneoplastic findings. 

To the best of our knowledge, the relationship between the absence of long-lasting morning stiffness (MS) at the time of diagnosis of PMR and the risk of neoplasia has been very scarcely investigated. In 2002, Niccoli et al. reported on three patients presenting PMR-like manifestations, such as the absence of prolonged MS, inefficacy of corticosteroids and absence of shoulder US pathological findings, which led to the diagnosis of renal cell carcinoma [8]. In 2021, Ramon et al. assessed the frequency of occult solid malignancy in patients with PMR-like findings using a standardized systematic thoracic, abdominal and pelvic computed tomography (CT) scan. They found no significant association between MS duration > 1 h and diagnosis of cancer (*p* = 0.68) [9]. Recently, we reported on a patient in whom the absence of long-lasting MS and the partial efficacy of GCs were the only atypical findings, and PMR was the onset of prostate cancer [10]. 

Therefore, we investigated whether and to what extent the absence of long-lasting MS at the time of diagnosis of PMR was related to the probability of diagnosing neoplasias during follow-up. 

## 2. Materials and Methods

We performed an observational, retrospective, single-center cohort study. The study was performed in Sant’ Agnello (health district no.59, Azienda Sanitaria Locale Napoli 3, Italy) with the approval of the internal Ethics Committee (identification number: SA-2022.4).

Inclusion criteria: We assessed all patients consecutively referred to our rheumatologic outpatient clinic between January 2015 and December 2020, who could be classified as PMR according to 2012 EULAR/ACR criteria. In particular, we assessed all patients scoring a minimum of 5 points with a combination of clinical and ultrasound (US) criteria. Two investigators (CM and AC) independently reviewed the records of these patients for the diagnosis of neoplasias during a two-year follow-up. The following variables were assessed: gender; age at onset; family history of malignancy; presence or absence of MS duration longer than 45 min; routine analyses; chest X-ray; abdominal US examination; body mass index (BMI); patient’s numerical visual analogue scale (VAS); standardized response to 12.5–15 mg/day of prednisolone (or prednisolone equivalent); duration of follow-up; type of neoplasia diagnosed during follow-ups; and PMR regression (or not) after treatment of neoplasia. In particular, MS > 45 min was collected in a dichotomous way (Yes/No); patient’s VAS was from 0 to 100. Routine analyses also included the rheumatoid factor (RF), anti-citrullinated protein antibodies (ACPA), anti-nuclear antibodies (ANA), anti-neutrophil citoplasmic antibodies (ANCA) and, in male patients, the serum prostate-specific antigen (PSA). Family history of cancer was assessed as first-degree familiarity. Finally, a positive response to prednisolone was a patient-reported pain improvement associated with the erythrocyte sedimentation rate (ESR) and C-reactive protein (CRP) improvement measure of 75% within a week, and the normalization of laboratory parameters within 4 weeks. 

Exclusion criteria were: (a) follow-up duration < two years; (b) malignancy prior to PMR; (c) first-degree familiarity of malignancy; (d) incomplete data; and (e) diagnostic change during follow-up in different rheumatologic diseases (for example, from PMR to seronegative elderly-onset rheumatoid arthritis). 

Statistical analysis: The descriptive data of normally distributed variables are reported as the mean ± standard deviation (SD), while binary data are reported as percent frequency. The differences between groups were compared using an unpaired Student’s *t*-test when clinical and biological data were expressed as continuous variables, and the χ^2^ test for categorical variables. Odd ratios for MS associated with neoplasia were calculated. Differences were assumed to be significant at two-tailed *p* values < 0.05. All the data were analyzed using a standard statistical package (SPSS for Windows version 21.0, Chicago, IL, USA). 

## 3. Results

We included 143 patients fulfilling the inclusion and exclusion criteria: 35 men and 108 women; a median age of 71.5 years; and 35 had MS duration < 45 min at the time of PMR diagnosis. We listed their main demographic, clinical and laboratory data in Table 1. In 10 patients (6.9%), a malignancy was diagnosed (Table 2); its diagnosis was always in the first 6 months of their follow-ups. Among these patients, seven did not have long-lasting MS. Among the remaining 133 patients without subsequent malignancy, 28 did not have long-lasting MS. The odds of cancer were 0.114 (95% C.I. 0.028, 0.471). The presence of MS duration < 45 min was inversely associated with the presence of neoplasia during follow-ups. Finally, in the group of patients without long-standing MS, who were analyzed according to the presence or absence of neoplasia, a lower response to GCs in the group without neoplasia was the only statistically different variable (71% vs. 100%; *p* < 0.0001, Table 3).

## 4. Discussion

To our best knowledge, this is the first study that specifically assessed the potential paraneoplastic value of long-lasting MS at the time of diagnosis of PMR.

Long-lasting MS is listed in all diagnostic and classification criteria proposed for PMR. However, patients did not easily report it. For example, these data were not available in 33 out of 125 patients with PMR in the 2012 European League against Rheumatism/American College of Rheumatology (EULAR/ACR) collaborative study [11]. 

In addition, MS duration was not unanimously determined in the published literature [12]. For example, MS duration was >45 min in the 2012 EULAR/ACR classification criteria. In the EULAR/ACR collaborative study, long-lasting MS had an OR of 5 when a combination of clinical and US criteria was used, and of 6.2 when only clinical criteria were used. These values were much higher than those attributed to all other listed criteria. Nevertheless, PMR categorization was possible even if long-lasting MS was absent, provided that all the other proposed criteria were satisfactorily met. Therefore, some of our patients could be classified as PMR in the absence of MS duration > 45 min, because their total score was 5 or more using clinical and US criteria. 

We found an inverse association between long-lasting MS at the time of diagnosis of PMR and the development of neoplasia. It is worth highlighting that the removal of the neoplastic mass led to a fast disappearance of clinical, US and laboratory findings in all eight patients diagnosed with solid cancers, thus supporting the diagnosis of paraneoplastic PMR [13,14]. The two patients diagnosed with myelodysplastic syndromes are still in follow-up. 

Our data suggested that the absence of long-lasting MS at the time of diagnosis of PMR is a paraneoplastic warning. An explanation to understand this finding is merely speculative. An occult neoplasia can promote the release of substances that interfere with MS duration in genetically predisposed patients, and this seems the most likely hypothesis. As proposed by some researchers, the correlation between PMR and cancer could be an altered immune response, and an increased release of some cytokines, such as interleukin-6 (IL-6), is common in both conditions [15,16]. Nothing is known concerning how and if these responses can cause or favor the absence of long-lasting MS in patients with PMR having an occult malignancy, so far. 

Some guidelines recommend simple screening investigations in patients with PMR to exclude other differential diagnoses, and cancer is among these [17,18]. More complex and expensive diagnostic investigations cannot instead be used for mass screening in patients with PMR because they are unsustainable in terms of cost-effectiveness. According to our study, the absence of long-lasting MS at the time of diagnosis of PMR may identify a subset of patients with PMR who should undergo a thorough investigation to exclude neoplasias. In this subset of PMR patients, more complex and expensive investigations can be cost-effective. 

In addition, as highlighted in Table 3, a positive response to GCs was present in 100% of the 28 PMR patients who did not have long-lasting MS at the time of diagnosis and no neoplasia during their follow-ups. On the contrary, a positive response to GCs was present in only 71% of PMR patients who did not have long-lasting MS and were diagnosed with neoplasia during follow-up. Among the variables we assessed, a positive response to GCs was the only one that was statistically significant (*p* < 0.0001). This suggests that an inadequate response to GCs in PMR patients who have MS duration < 45 min at the time of diagnosis should lead to increased investigation to exclude neoplasia. Although this was not the aim of our study, this point should be taken into account in everyday clinical practice. 

Our study has some limitations. First of all, we assessed MS duration in a dichotomous way (Yes/No). This did not allow us to assess whether the paraneoplastic value of the absence of long-lasting MS at the time of diagnosis was related to its time in minutes. Secondly, our study was a single-center study. Therefore, multi-center studies are needed to confirm our results. 

Finally, our study was a retrospective study and may have all the limitations of this type of study. We hope that our inclusion and exclusion criteria reduced most of the potential bias. In particular, we systematically asked all the patients with suspicion of PMR attending our rheumatologic outpatient clinic whether they had MS [19,20,21]. Therefore, we had no bias in the collection of these data. In addition, all enrolled patients had a minimum follow-up of two years, as this is the proposed maximum timeframe for occult neoplasia to become clinically apparent when paraneoplastic findings are present in patients with PMR [22,23,24]. 

## 5. Conclusions

The absence of long-lasting MS does not in itself exclude PMR categorization, provided that all the other proposed classification criteria are satisfactorily met. 

However, this finding may be a paraneoplastic warning. Therefore, in this subset of PMR patients, further diagnostic investigations should be performed to exclude this possibility before idiopathic PMR is diagnosed and the treatment with GCs is initiated.

The ways in which occult neoplasia may affect MS in patients classified as having PMR are still speculative, and should be addressed in future research studies. 

## Figures and Tables

**Table 1:** Main demographic, laboratory and clinical data in all enrolled patients, according to presence or absence of long-lasting morning stiffness (MS).

	All (143)	MS, Yes (108)	MS, No (35)	*p*
Age at diagnosis, years	71.58 ± 7.12	71.43 ± 7.00	72.05 ± 7.57	0.655
Female, %	83.9%	85.2%	80%	0.472
CRP, mg/L	16.59 ± 7.58	15.32 ± 7.07	20.49 ± 7.87	**<0.0001**
ESR, mm/h	59.48 ± 16.59	56.90 ± 16.19	67.42 ± 15.45	**<0.001**
patient’s VAS	76.01 ± 14.49	77.13 ± 13.18	72.57 ± 17.71	0.106
positive response to GCs	96.5%	100%	85.7%	**<0.0001**
neoplasia	10 (6.9%)	3 (2.7%)	7 (20%)	**<0.0001**

Data are expressed as mean ± standard deviation or percentual. MS = morning stiffness; CRP = C reactive protein; ESR = erythrocyte sedimentation rate; VAS = visual analogic scale; GCs = glucocorticoids.

**Table 2:** Malignancies diagnosed during follow-up.

NEOPLASIA	N.
Breast	3
Kidney	2
Lung	1
Prostate	1
Colon	1
Myelodysplastic syndromes	2

**Table 3:** Main demographic, laboratory and clinical data in patients with absence of long-lasting morning stiffness, according to presence or absence of neoplasia.

	All (35)	Neoplasia (7)	No Neoplasia (28)	*p*
Age at diagnosis, years	72.05 ± 7.57	71.35 ± 7.56	74.85 ± 49	0.106
Female, %	80%	71.4%	82.1%	0.217
CRP, mg/L	20.49 ± 7.87	19.28 ± 8.38	20.78 ± 7.88	0.300
ESR, mm/h	67.42 ± 15.45	67.85 ± 13.17	67.32 ± 16.19	0.093
patient’s VAS	72.57 ± 17.71	68.57 ± 19.51	73.57 ± 17.47	0.113
positive response to GCs	85.7	71%	100%	**<0.0001**

Data are expressed as mean ± standard deviation or percentual. CRP = C reactive protein; ESR = erythrocyte sedimentation rate; VAS = visual analogic scale; GCs = glucocorticoids.

## Data Availability

The data used to support the findings of this study are available from the corresponding author upon request.
